# Supplementary material for: Longitudinal IgA and IgG Response, and ACE2 Binding Blockade, to Full-Length SARS-CoV-2 Spike Protein Variants in a Population of Black PLWH Vaccinated with ChAdOx1 nCoV-19
Source: Viruses. 2023 Feb 6;15(2):448. doi: 10.3390/v15020448 (PMC9965153; doi:10.3390/v15020448)
Supplement: Supplementary file 1 [file viruses-15-00448-s001.zip › viruses-2152312-supplementary.pdf]

Materials and Methods

Printing S variant protein microarrays

A total of 40 µl crude protein lysate was transferred to a 384-well V-bottom plate (Genetix). The plate was centrifuged (Megafuge 40R, Thermo Scientific) for 2 minutes at 1200 ×g at 4°C before starting the microarray print run. The microarrays were printed in 24-plex on a streptavidin-coated glass slide (Nexterion, Schott) using the QArray2 robotic array (Genetics, Berkshire, UK) equipped with eight 300 µm flat-tipped solid pints. Printing occurred at 18°C at 60% humidity. Each array was printed with the following settings: Ink time = 500 ms, microarray pattern = 3 × 3, 800 µM spacing, maximum stamps per ink = 1, number of stamps per spot = 1, printing depth = 150 µm, water washes = 60 seconds, ethanol washes = 10 seconds, and 1 second drying. Post-printing, the slides was blocked (25 mM HEPES (MERCK), 50 mM KCl (MERCK), 20% glycerol (MERCK), 0.1% Triton X-100 (MERCK), 1 mM DTT (MERCK) and 50 µM biotin (Glentham Life Sciences)), washed 2x 5 minutes with *Wash buffer* (PBS (source?) and 0.2% Tween-20 (MERCK)) and 2x 5 minutes wash in PBS only. The slides were subsequently stored at -20°C in storage buffer (60% glycerol in PBS) until used in assays.

Supplementary Figures

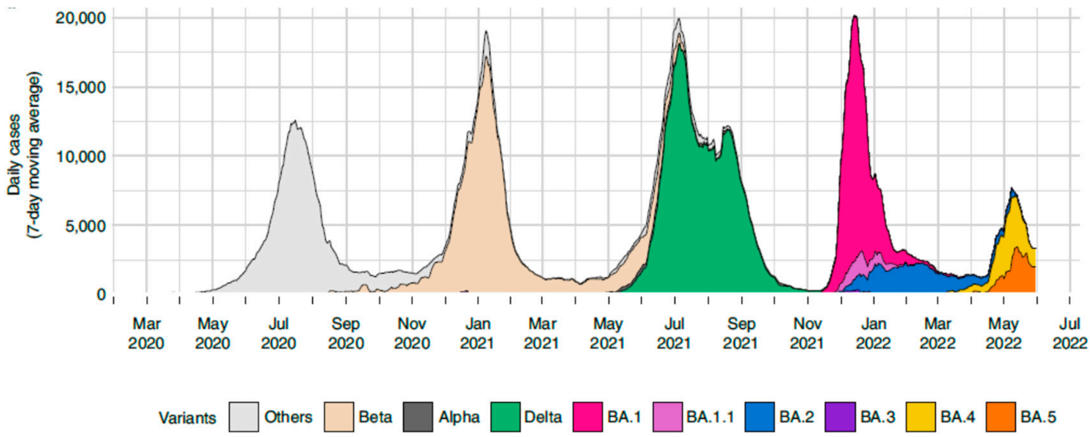

Supplementary Figure S1. Distribution of SARS-CoV-2 lineages in South Africa. The progression of the 7-day rolling average of daily reported case numbers in South Africa over 2 years of the pandemic (April 2020 to May 2022). Daily cases are coloured by the inferred proportion of ASRS_CoV-2 variants prevalent at a particular period in the pandemic (Adapted from Tegally et al, 2022). Sample were collected 17 August 2020 and 12 November 2020.

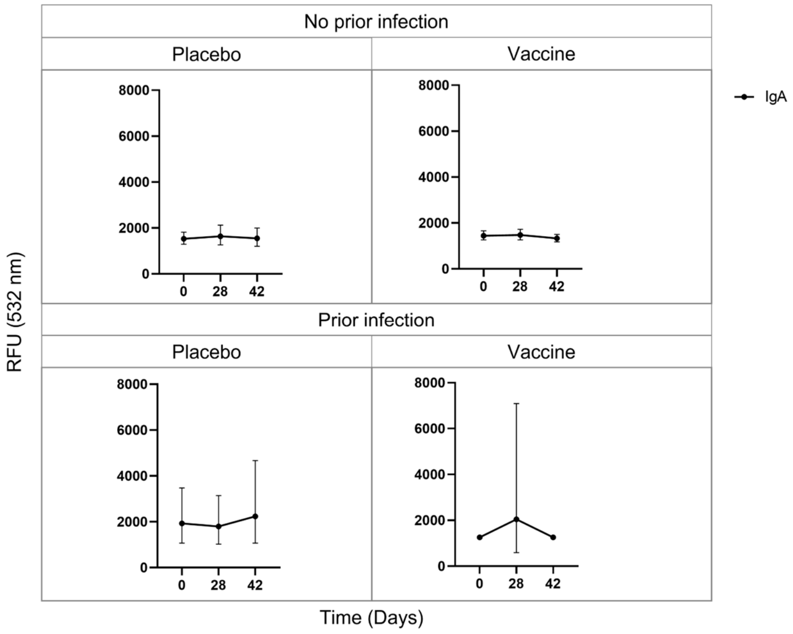

Supplementary Figure S2. Longitudinal IgA towards SARS-CoV-2 spike protein in HIV-negative participants. The graphs depict the longitudinal change in IgA against the SARS-CoV-2 ancestral spike (S) protein in response to the ChAdOx1 nCoV-19 vaccinated in HIV-negative participant. The average RFU is represented and the error bars indicate the minimum and maximum values.

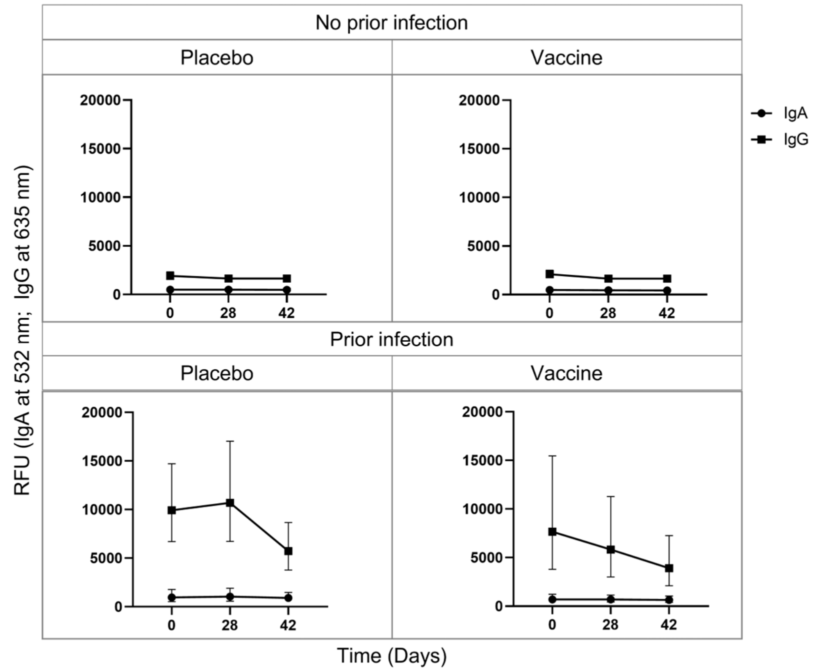

Supplementary Figure S3. Longitudinal IgA and IgG towards SARS-CoV-2 nucleocapsid protein. The graphs depict the longitudinal change in the IgA and IgG against the SARS-CoV-2 nucleocapsid protein in response to the ChAdOx1 nCoV-19 vaccinated in people living with HIV (PLWH). Participants were assessed by subdividing into those with or without a previous infection, and whether they received the vaccine or placebo.

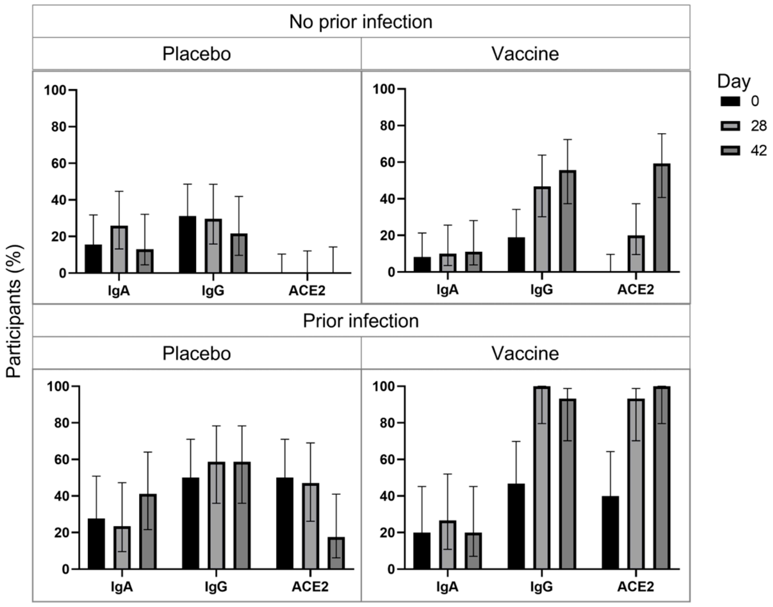

Supplementary Figure S4. Longitudinal IgA and IgG response, and ACE2 binding blockade to SARS-CoV-2 spike protein. The graphs depict the longitudinal (Day 0, Day 28 and Day 42) change in the percentage of people living with HIV (PLWH) with an IgA and IgG response, and ACE2 binding blockade,

to the SARS-CoV-2 ancestral spike protein. Participants were assessed by subdividing into those with or without a previous infection, and whether they received the vaccine or placebo.

Supplementary Tables

Table S1. Demographics for people living with HIV (PLWH).

Variable	Placebo arm		Vaccine arm	
	No prior infection	Prior infection	No prior infection	Prior infection
N enrolled	33	18	37	15
Female n (%)	25 (75.8)	16 (88.9)	25 (67.6)	11 (73.3)
Male n (%)	8 (24.2)	2 (11.1)	12 (32.4)	4 (26.7)
Median Age in years (IQR)	40 (36-46)	43 (40-47)	35 (32-45)	42 (31-47)
BMI n(%)				
Underweight	1 (3%)	0 (0%)	3 (8.1%)	1 (6.7%)
Normal	12 (36.4%)	5 (27.8%)	16 (43.2%)	8 (53.3%)
Overweight	11 (33.3%)	6 (33.3%)	14 (37.8%)	3 (20%)
Obese	9 (27.3%)	7 (38.9%)	4 (10.8%)	3 (20%)
Smoker	11 (33.3)	4 (22.2)	14 (37.8)	4 (26.7)
Alcohol	10 (30.3)	10 (55.6)	17 (45.9)	9 (60)
Health worker	1 (3)	0 (0)	1 (2.7)	0 (0)
Race n(%)				
Black	33 (100%)	18 (100%)	36 (97.3%)	15 (100%)
Coloured	0 (0%)	0 (0%)	0 (0%)	0 (0%)
White	0 (0%)	0 (0%)	1 (2.7%)	0 (0%)
Other	0 (0%)	0 (0%)	0 (0%)	0 (0%)
Hypertension	2 (6.1)	5 (27.8)	4 (10.8)	0 (0)
Respiratory system	7 (21.2)	3 (16.7)	3 (8.1)	3 (20)
Diabetes HbA1c level				
Low	3 (9.1%)	2 (11.1%)	3 (8.1%)	2 (13.3%)
Normal	30 (90.9%)	16 (88.9%)	33 (89.2%)	13 (86.7%)
High	0 (0%)	0 (0%)	1 (2.7%)	0 (0%)
Median CD4 count (IQR)	593 (498-900)	704 (442-865)	746 (554-952)	696 (521-946)
Median CD4 percent (IQR)	34 (29-39)	37 (28-40)	36 (32-41)	37 (33-40)
Median detectable Viral load (IQR)	10 (10-26)	10 (10-28)	30 (10-96)	46 (28-94)
VL < 50 n (%)	12 (80)	6 (85.7)	7 (63.6)	2 (66.7)
ART				
Boosted PI + 1 NRTI	2 (7.4%)	1 (9.1%)	1 (3.4%)	0 (0%)
Boosted PI +2 NRTIs	2 (7.4%)	0 (0%)	1 (3.4%)	0 (0%)
INSTI + 2 NRTIs	4 (14.8%)	1 (9.1%)	5 (17.2%)	1 (14.3%)
NNRTI +2 NRTIs	19 (70.4%)	9 (81.8%)	22 (75.9%)	6 (85.7%)
Time on ART				
<1 year	3 (11.1%)	1 (9.1%)	3 (10.3%)	2 (28.6%)
1-<5 years	9 (33.3%)	3 (27.3%)	12 (41.4%)	4 (57.1%)
>=5 years	15 (55.6%)	7 (63.6%)	14 (48.3%)	1 (14.3%)
Median time between doses in days (IQR)	28 (27-28)	28 (25-28)	28 (23-28)	28 (24-28)
Median time post-booster in days (IQR)	14 (14-14)	14 (14-14)	14 (14-14)	14 (14-14)
